# Derivation of intermediate to silicic magma from the basalt analyzed at the Vega 2 landing site, Venus

**DOI:** 10.1371/journal.pone.0194155

**Published:** 2018-03-27

**Authors:** J. Gregory Shellnutt

**Affiliations:** National Taiwan Normal University, Department of Earth Sciences, Taipei, Taiwan; Heidelberg University, GERMANY

## Abstract

Geochemical modeling using the basalt composition analyzed at the Vega 2 landing site indicates that intermediate to silicic liquids can be generated by fractional crystallization and equilibrium partial melting. Fractional crystallization modeling using variable pressures (0.01 GPa to 0.5 GPa) and relative oxidation states (FMQ 0 and FMQ -1) of either a wet (H_2_O = 0.5 wt%) or dry (H_2_O = 0 wt%) parental magma can yield silicic (SiO_2_ > 60 wt%) compositions that are similar to terrestrial ferroan rhyolite. Hydrous (H_2_O = 0.5 wt%) partial melting can yield intermediate (trachyandesite to andesite) to silicic (trachydacite) compositions at all pressures but requires relatively high temperatures (≥ 950°C) to generate the initial melt at intermediate to low pressure whereas at high pressure (0.5 GPa) the first melts will be generated at much lower temperatures (< 800°C). Anhydrous partial melt modeling yielded mafic (basaltic andesite) and alkaline compositions (trachybasalt) but the temperature required to produce the first liquid is very high (≥ 1130°C). Consequently, anhydrous partial melting is an unlikely process to generate derivative liquids. The modeling results indicate that, under certain conditions, the Vega 2 composition can generate silicic liquids that produce granitic and rhyolitic rocks. The implication is that silicic igneous rocks may form a small but important component of the northeast Aphrodite Terra.

## Introduction

Silicic magma (SiO_2_ > 60 wt.%) on Earth is produced by differentiation (fractional crystallization) of mafic (basalt, basaltic andesite) magma, partial melting of crustal lithologies, and/or hybrid processes of assimilation and fractional crystallization [1–5]. Most silicic magmas are primarily produced at subduction zones and collisional settings but minor volumes are produced at extensional settings, including large igneous provinces, oceanic ridge settings, and continental rifts [6–9]. Consequently, silicic igneous rocks (dacite, granodiorite, rhyolite, granite) are mostly associated with crustal recycling processes and ubiquitous within the continental crust of Earth but less so within oceanic crust.

The occurrence of sialic (**si**lica and **al**umina-rich rocks) crust is a defining characteristic of Earth with respect to other telluric planets, satellites and asteroids in the Solar System [10–12]. The surface of Venus is dominated by vast volcanic plains with subordinate highland terranes and volcanic (cones, calderas) edifices [13–15]. Although there are distinct (pancake domes) types of volcanic edifices on Venus that are not common on Earth, the morphology of many volcanic flow fields appears to be analogous to terrestrial pahoehoe and a’a flows that are typical of shield volcanoes (Hawaii) and some terrestrial flood basalt provinces [16,17]. There is also evidence for volatile-rich pyroclastic deposits, an eruption style associated with Plinian eruptions and subduction zone settings on Earth [18,19]. The surface composition of Venus was measured at seven locations. The full suite of major elements (except Na) of Venusian rocks are reported for the Venera 13, Venera 14 and Vega 2 landing sites whereas only K, U and Th concentrations were reported from the remaining (Vega 1, Venera 8, Venera 9 and Venera 10) locations [20–23]. The available geochemical data indicates that the most common rock type encountered at the landing sites was basalt. However, the Venera 8 rock, based on K_2_O (K_2_O = 4.8 ± 1.4 wt%), U (U = 2.2 ± 0.7 ppm) and Th (Th = 6.5 ± 0.2 ppm) contents, is interpreted to be similar to rhyolite, monzonite or leucitite and may represent a rock that is typical of terrestrial continental crust [23,24].

Earth-style plate tectonics is not operating on Venus and there is limited evidence that subduction zone systems existed in the geological past but there are a number of studies that suggest silicic volcanic and plutonic rocks may be present on Venus [19,24–33]. The atmosphere of Venus is known to contain water vapour (30 ± 15 ppm), SO_2_ (150 ± 30 ppm) and HCl (0.6 ± 0.12 ppm) that may be related to volcanic degassing suggesting the mantle may contain abundant volatiles [34–36]. Climate simulations and the high deuterium-to-hydrogen ratio (150 ± 30 times that of terrestrial water) of the atmosphere suggest that Venus may have had significant quantities of surface water. Therefore it is possible that primary melts from the Venusian mantle and/or crustal lithologies contained sufficient quantities of volatile elements (H_2_O, CO_2_, Cl, F) that could lead to the formation of silicic magmas by high degrees of fractional crystallization or partial melting [1,37–39]. Moreover, petrological processes (partial melting and fractional crystallization) and magma conditions (relative oxidation state, pressure, volatile content) that form silicic magmas at non-subduction-related settings (rift zones) are not restricted to Earth and therefore silicic igneous rocks should be present on Venus [28,40,41].

Previous petrological modeling using the compositions from the Venera 13 and Venera 14 landing sites demonstrated that it is possible to produce silicic compositions derived from the basalt of the volcanic plains [31]. Unlike the Venera 13 and Venera 14 probes that landed on the volcanic plains (western Navka Planitia), Vega 2 landed on the northeastern (NE) flank of Aphrodite Terra [27]. Aphrodite Terra is the largest highland terrane of Venus and shows evidence of regional-scale deformation [42–44]. The purpose of this study is to determine if silicic (SiO_2_ > 60 wt%) magma can be generated from a parental magma or rock similar in composition to the basalt analyzed at the Vega 2 landing site. The petrological software Rhyolite-MELTS is used to assess if fractional crystallization and/or partial melting under reasonable geological conditions (pressure, relative oxidation state, water content) can yield liquid compositions that are similar to terrestrial silicic (rhyolite, granite) rocks. The possible presence of silicic rocks has significant implications on the geologic structure of NE Aphrodite Terra but also for the crustal structure of highland terranes across Venus.

## Surface composition of Venus

The Vega 2 landing site is located along the southeastern edge of Rusalka Planitia on the northeastern slope of Aphrodite Terra. The landing site was selected in order to determine if there is a compositional difference between rocks from the highland and lowland regions of Venus [22,23]. The rock analyzed at the Vega 2 landing site is somewhat compositionally similar to the Venera 14 site but they are noticeably different with respect to CaO, TiO_2_ and SO_3_ contents (Table 1). The rock is described as an olivine gabbro-norite or as normal mid-ocean-ridge basalt (N-MORB) [22,23]. However, the Vega 2 composition has higher MgO and lower CaO, TiO_2_ and FeOt than MORB [45] and is more similar to the range of basalt compositions found within continental flood basalt provinces. In comparison, the Venera 13 and 14 landing sites are located near western Navka Planitia, SSE of Beta Regio, and correspond to surface morphology of upland rolling plains and flat lowland [21,23]. The rocks analyzed at the Venera landing sites may be different as the Venera 13 site appears to be an alkaline (phonolitic tephrites or mafic leucitic) mafic rock whereas the rock at the Venera 14 site is tholeiitic basalt similar to terrestrial mid-ocean ridge basalt [21].

**Table 1:** Major element compositions of basalt from Venus and the compositions used for modeling.

Sample	Venera 13 [21]	Venera 14 [21]	Vega 2 [22]	Venera 8 [24] (inferred)	Vega 2 (anhydrous)	Vega 2 (hydrous)	Vega 2 (adjusted)^†^	Vega 2 (adjusted)^+^
SiO_2_ (wt.%)	45.1 ± 3.0	48.7 ± 3.6	45.6 ± 3.2	58.3–65.6	50.25	50.00	51.61	51.35
TiO_2_	1.6 ± 0.45	1.25 ± 0.41	0.20 ± 0.1	0.5–1.5	0.22	0.22	0.23	0.23
Al_2_O_3_	15.8 ± 3.0	17.9 ± 2.6	16.0 ± 1.8	13.4–16.2	17.63	17.54	18.11	18.02
FeO	9.3 ± 2.2	8.8 ± 1.8	7.7 ± 1.1	3.2–6.8	8.49	8.44	8.72	8.67
MnO	0.2 ± 0.1	0.16 ± 0.08	0.14 ± 0.12	0.1–0.2	0.15	0.15	0.16	0.16
MgO	11.4 ± 6.2	8.1 ± 3.3	11.5 ± 3.7	1.6–4.1	12.67	12.61	10.31	10.26
CaO	7.1 ± 1.0	10.3 ± 1.2	7.5 ± 0.7	2.8–6.4	8.27	8.22	8.49	8.45
Na_2_O	2.0 ± 0.5*	2.4 ± 0.4*	2.0 ± 0.5*	2.5–4.4	2.20	2.19	2.26	2.25
K_2_O	4.0 ± 0.6	0.2 ± 0.07	0.1 ± 0.08	3.4–4.9	0.11	0.11	0.11	0.11
P_2_O_5_				0.2–0.70				
SO_3_	1.6 ± 1.0	0.88 ± 0.77	4.7 ± 1.5		-	-	-	-
Cl	< 0.3	< 0.4	< 0.3		-	-	-	-
H_2_O					-	0.5	-	0.5
Total	98.1	98.7	95.4		100	100	100	100

*The Na_2_O content is calculated for the Venera 13, 14 and Vega 2 data [21, 22]. The Vega 2 (adjusted) composition assumes kieserite (MgSO_4_·H_2_O) was removed from the original composition. See text for details.

† = anhydrous.

+ = hydrous.

## Modeling conditions

Thermodynamic modeling software Rhyolite-MELTS is a useful tool to evaluate the petrological evolution of silicate magma systems [46]. Rhyolite-MELTS (version 1.0.1.) is calibrated to model the evolution of silicate liquids that fall within the compositional range of nearly all igneous rocks. The software is specifically optimized for silicic magma systems and allows the user to modify intrinsic thermodynamic parameters such as relative oxidation state (*f*O_2_), pressure (GPa) and water (wt%) content of the system that is being modeled. Constraining the petrological conditions for the formation of Venusian basalt is important for selecting the optimal modeling conditions.

The modeling results are compared to terrestrial andesite and rhyolite compiled from the GEOROC database (georoc.mpch-mainz.gwdg.de/georoc/Entry.html). The data were selected according to the following criteria: 1) LOI (loss on ignition) < 2.5 wt%, 2) ferroan (FeOt/FeOt+MgO > 0.70) composition, and 3) andesite is defined as SiO_2_ > 52 wt% but < 64 wt% and the rhyolite is defined as SiO_2_ > 69 wt% (S1 Table). Andesitic rocks are commonly associated with subduction zone settings but tend to be magnesian (FeOt/FeOt+MgO < 0.70) in composition. Although ferroan compositions are selected, it does not preclude the possibility that ferroan andesite was generated at a subduction zone setting. Ferroan rhyolite, on the other hand, is frequently associated with anorogenic tectonic settings [47]. The total range of data and 95% confidence fields are plotted together on each figure.

### Composition

A number of studies have modelled the petrogenesis of the Venusian basalts [15,23,38,48,49]. It is thought that the basalts were produced by processes similar to Earth, specifically, by partial melting and fractionation from both high (> 1.8 GPa) pressure (Venera 13) and low (< 0.2 GPa) pressure (Venera 14 and Vega 2) conditions. Some of the models imply that there could be significant concentrations (~0.2 wt%) of water within the mantle. Furthermore the Venusian basalt compositions suggest that the thermal regime required to produce the primary melts was probably close to ambient (~1400°C) mantle potential temperatures (*T*_P_) of Earth however there are suggestions that the mantle thermal conditions for Vega 2 basalt could be significantly (~1780°C) higher and closer to thermal regime of terrestrial Archean komatiites [48,50–53].

For this study, the basalt measured at the Vega 2 landing site is used to determine if silicic compositions can be generated either by fractional crystallization (anhydrous and hydrous) or equilibrium partial melting (anhydrous and hydrous). However, there is uncertainty regarding the exact nature of the Vega 2 composition as it contains a high concentration of SO_3_ (SO_3_ = 4.7 ± 1.5 wt%) and therefore may be representative of soil or a mixture of soil and rock. Consequently, two bulk rock compositions are used for modeling (Table 1).

The first composition is the SO_3_-free (volatile-free) Vega 2 basalt normalized to 100%. The assumption is that the SO_3_ is partitioned into non-silicate minerals such as sulphide or sulphate minerals at various proportions. However, it is unlikely that the SO_3_ is exclusively hosted within an Fe-rich sulphide mineral (e.g. pyrite, pyrrhotite, marcasite) given the relatively low bulk FeO content (FeOt = 7.7 ± 1.1 wt%) of the rock. For example if the sulphur (SO_3_ = 4.7 ± 1.5 wt%) is hosted within pyrite (FeS_2_) or pyrrhotite (Fe_1-X_S) then 40–80% (3–6 wt%) of the total FeO of the sample is derived from Fe-sulphide minerals. Furthermore, it also unlikely that the SO_3_ component is derived from anhydrite (CaSO_4_) or gypsum (CaSO_4_·2H_2_O). If the soil component was exclusively derived from anhydrite or gypsum then it would represent ~45% (~3.2 wt%) of the total CaO of the rock (CaO = 7.5 ± 0.7 wt%). It is possible that the SO_3_ is hosted within Fe-poor sulphide minerals such as millerite (NiS), chalcocite (Cu_2_S), covellite (CuS) or digenite (Cu_9_S_5_) or within sulphate minerals such as barite (BaSO_4_), kieserite (MgSO_4_·H_2_O), and celestine (SrSO_4_).

The second composition assumes the SO_3_ is hosted by a magnesium sulphate mineral, specifically kieserite (MgSO_4_·H_2_O) or its anhydrous equivalent. It is possible that the relatively high concentration of MgO (11.5 wt%) in the Vega 2 basalt could be due to the addition of MgO. Unlike other scenarios involving the computational removal of CaO or FeO in the Vega 2 rock, the resultant composition after adjusting the MgO is still within the range of basalt. Moreover, it is known that magnesium sulphate minerals exist on Mars and possibly Ceres [54,55]. If the high SO_3_ content is related to the presence of magnesium sulphate, then the bulk composition can be recalculated according to the amount of sulphate needed to explain the sulphur content in the sample. Assuming kieserite (MgO = 29.13 wt%; H_2_O = 13.02; SO_3_ = 57.86 wt%) is the source of the sulphur then MgO would decrease by ~2.4 wt% if it is removed from the rock (SO_3_ = 0.082*57.86 wt% = 4.74 wt%).

There are also uncertainties with respect to bulk Na_2_O content of the rock as it was not determined by X-ray fluorescence but was calculated and therefore is an approximation [22,23,56]. Furthermore, Rhyolite-MELTS does not necessarily introduce further uncertainty in the data but it is likely that phase equilibrium uncertainties will be produced because the program is based on experimental results.

### Pressure

The fractional crystallization and equilibrium partial melting models were conducted using pressures of 0.01 GPa, 0.1 GPa and 0.5 GPa. The selected pressures correspond to conditions expected on Venus for a lava lake (surface) setting (0.01 GPa), hypabyssal-plutonic setting (0.1 GPa) and a deep-seated plutonic setting (0.5 GPa).

**Fig 1:**
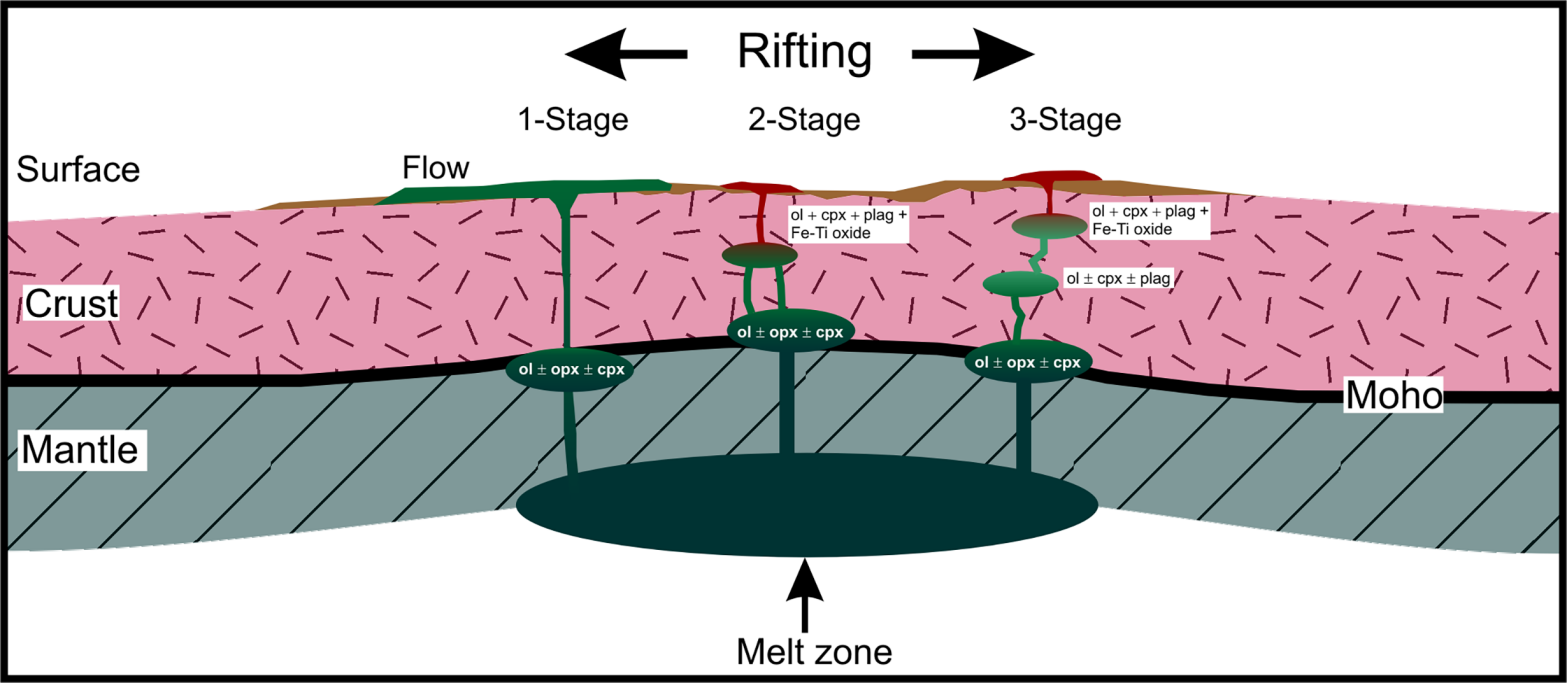
Conceptual crystallization scenarios of the parental magma of the Vega 2 rock. The 1-Stage differentiation scenario is deep seated fractionation primarily of olivine (ol) but may also include orthopyroxene (opx) and clinopyroxene (cpx). The 2-Stage scenario follows the 1-Stage scenario but the residual liquid stalls in the middle to upper crust and continues to differentiate into a silicic liquid due to fractionation of olivine (ol), clinopyroxene (cpx), plagioclase (plag) and Fe-Ti oxide minerals (ilmenite and magnetite). The 3-stage scenario follows the 1-Stage scenario of differentiation but has two steps of fractionation in order to generate a silicic residual liquid. The first step is fractionation of olivine (ol), clinopyroxene (cpx) and plagioclase (plag) at an intermediate depth in the middle crust or lowermost upper crust. The residual liquid then leaves the magma chamber and stalls in the upper crust and continues to differentiate by fractionating clinopyroxene (cpx), plagioclase (plag), Fe-Ti oxide minerals (ilmenite and magnetite) and possibly olivine (ol) before producing a silicic residual liquid.

Pressure is an important parameter for the genesis of silicic magmas by fractional crystallization. Numerical modeling, geological, and seismic studies indicate that mafic magmas undergo polybaric differentiation within the crust before eruption [57–64]. Assuming that either of the Vega 2 compositions used for the modeling is a close approximation of the silicate liquid that erupted, then the CaO content is too low and the Al_2_O_3_ is too high for a primary melt [65]. Consequently, it is very likely that the Vega 2 ‘liquid’ already experienced fractionation at some depth before it reached the surface [48,51]. Therefore the first models represent two stage polybaric crystallization (2-Stage) sequences in the sense that the primary melt was derived by partial melting of the mantle followed by fractionation of olivine ± orthopyroxene ± clinopyroxene (1-Stage) at an unknown depth (Fig 1). The second stage of fractionation is represented by the models at 0.01 GPa, 0.1 GPa and 0.5 GPa. In addition to the two stage models, three stage models (3-Stage) were calculated using the 65% (SO_3_-free model) and 70% (kieserite-adjusted model) liquid composition at 0.5 GPa for both compositions. After 35% and 30% crystallization of the parental magmas, the liquid compositions were then fractionated at a pressure of 0.1 GPa.

### Relative oxidation stage and initial water content

The relative oxidation state of the Vega 2 basalt liquid is unknown but, based on the compositional similarity to within-plate tholeiitic basalt, may range from the FMQ (fayalite-magnetite-quartz) buffer to the WM (wüstite-magnetite) buffer [66,67]. Therefore a relative oxidation state equal to the FMQ (fayalite-magnetite-quartz) buffer was used for both anhydrous (H_2_O = 0 wt%) and hydrous (H_2_O = 0.5 wt%) conditions. Models were run at FMQ -1 to test the effects of a reducing relative oxidation state in the hydrous fractionation models. The amount of water (H_2_O = 0.5 wt%) selected for the hydrous models is within the range tholeiitic basalt from Hawaii [68].

## Modeling results of Vega 2 basalt

### Fractional crystallization models (SO_3_-free)

**Fig 2:**
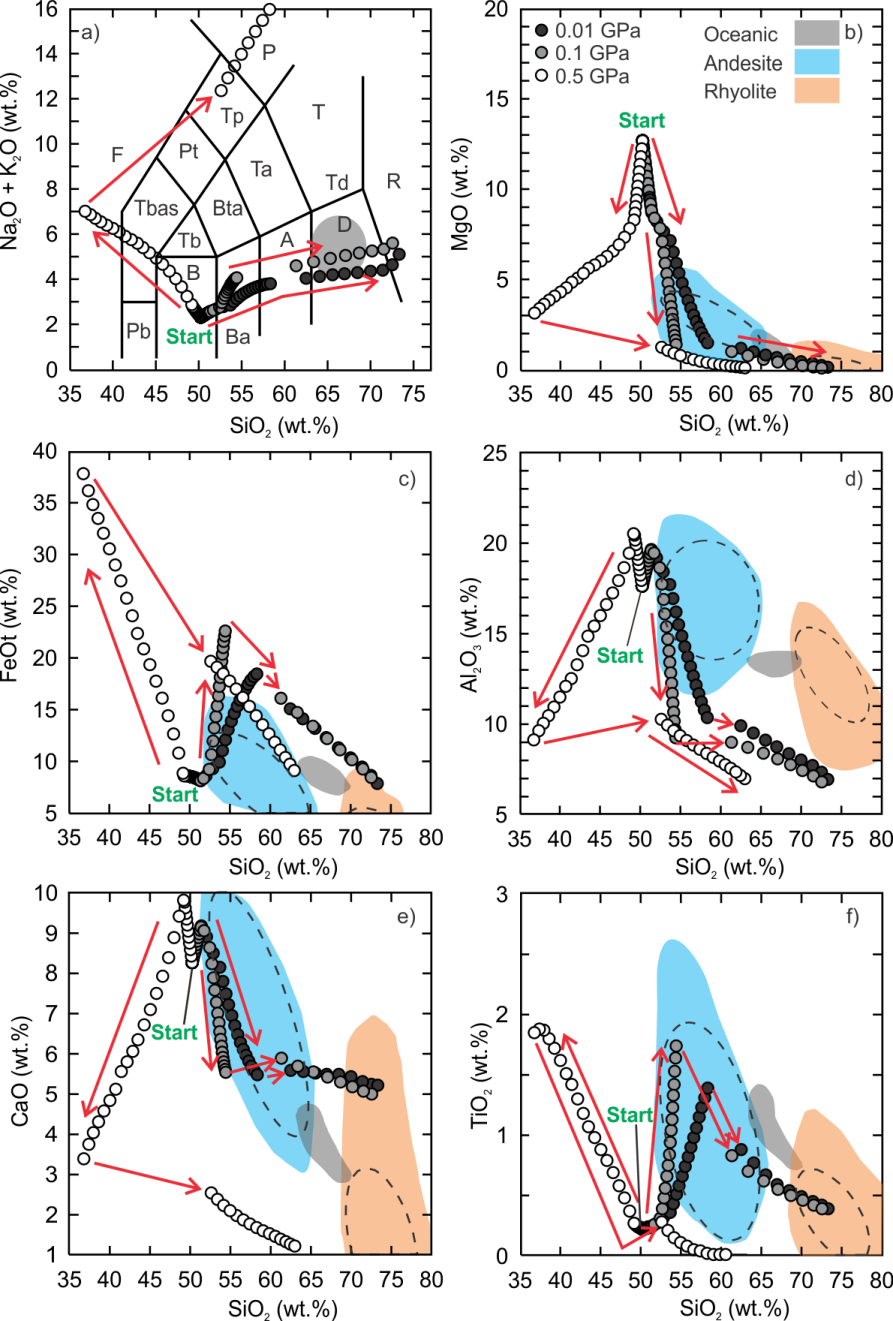
Results of Vega 2 anhydrous fractional crystallization models. Andesite (blue field) and rhyolite (red field) data (S1 Table) are compiled from the GEOROC database (georoc.mpch-mainz.gwdg.de/georoc/Entry.html). The grey field is the range of silicic rocks from a mid-oceanic ridge setting [8]. All data are normalized to 100%. The calculated 95% confidence ellipses (dashed) are added to the fields of terrestrial andesite and rhyolite. Panel a is the classification scheme of volcanic rocks [69]. F = foidite, Pb = picro-basalt, B = basalt, Ba = basaltic andesite, A = andesite, D = dacite, R = rhyolite, T = trachyte (quartz < 20%), Td = trachydacite (quartz > 20%), Ta = trachyandesite, Bta = basaltic trachyandesite, Tb = trachybasalt, TBas = tephrite (olivine < 10%) or basanite (olivine > 10%), Pt = phonotephrite, Tp = tephriphonolite, P = phonolite. Arrow is the direction of liquid evolution.

The fractionation models of the Vega 2 basalt demonstrate that a wide range of intermediate to silicic liquid compositions can be generated (S2 and S3 Tables). The low (0.01 GPa) and intermediate (0.1 GPa) pressure anhydrous models produced liquid compositions from basaltic andesite to rhyolite (Fig 2). The anhydrous silicic (SiO_2_ ≥ 70 wt%) liquids are within the range of terrestrial ferroan rhyolites at temperatures between 1010°C and 1050°C. The low pressure models indicate that the residual silicic liquids represent 9.5% to 12% of the total volume of magma whereas the intermediate models indicate the residual liquid is 6.6% to 8.7% of the initial magma volume. The Al_2_O_3_ content of the low and intermediate pressure models tends to be lower than terrestrial ferroan rhyolites and is related to the anhydrous nature of the model. Due to the prevalence of water within the mantle and crust of Earth it is unlikely that magmas could be 100% dry thus it is not surprising that the anhydrous high-SiO_2_ modelled compositions do not completely match with terrestrial rhyolitic rocks. The high pressure models produced alkaline compositions that range from basanite to phonolite but the SiO_2_ content is < 65 wt.% and the TiO_2_ is very low (< 0.10 wt.%).

**Fig 3:**
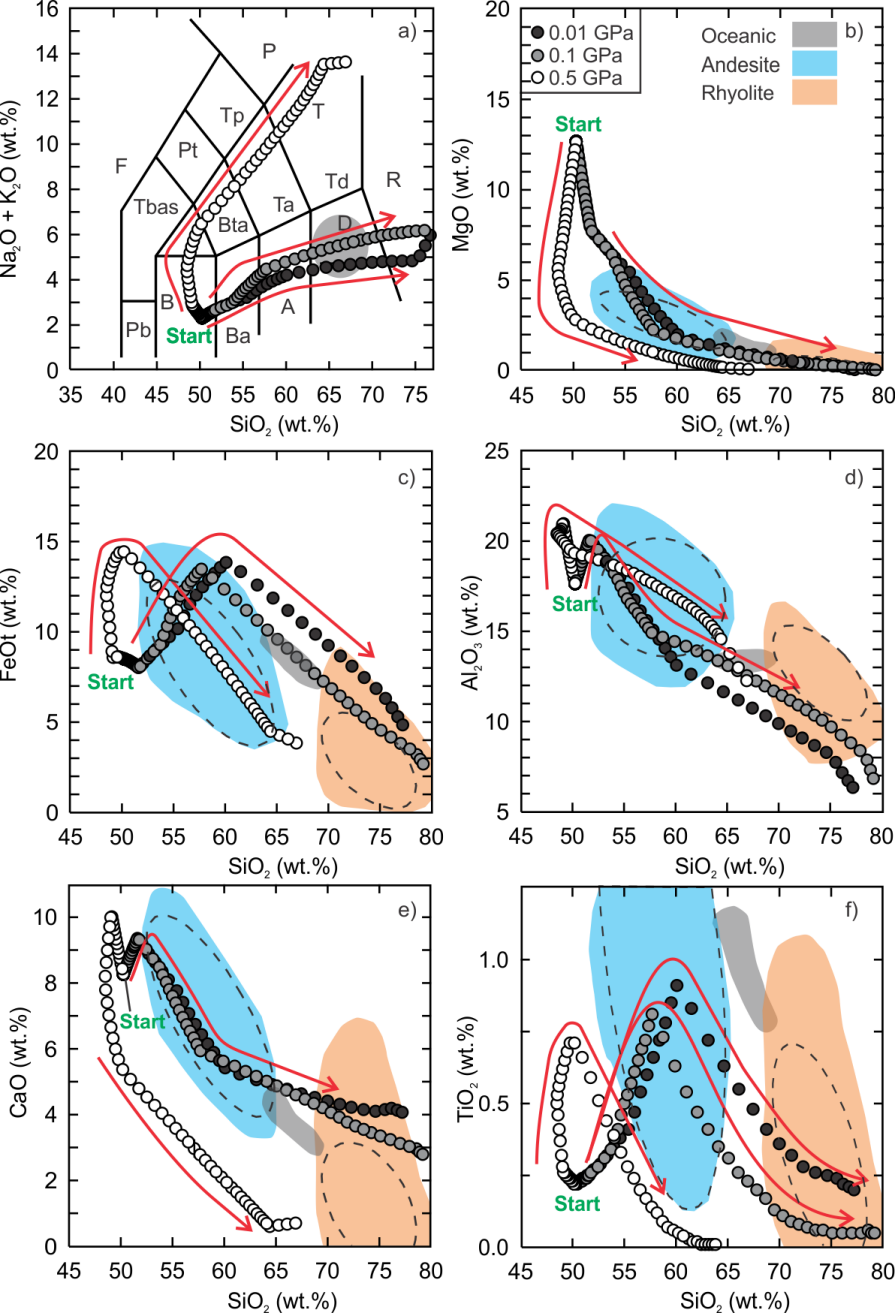
Results of Vega 2 hydrous (0.5 wt.% H_2_O) fractional crystallization models. The details of the figure are the same as Fig 2.

The low and intermediate pressure hydrous fractional crystallization models produced similar liquid compositions as the anhydrous models except that all major elements unambiguously fall within the terrestrial ferroan rhyolite field between 70 wt% and 75 wt% SiO_2_, and a silica gap was not produced (Fig 3). The bulk compositions of the silicic liquids (SiO_2_ > 65 wt%) have alumina saturation indices (molecular Al^3+^/Ca^2+^+Na^+^+K^+^)< 1 and range from metaluminous (Na^+^+K^+^/Al^3+^ < 1) to peralkaline (Na^+^+K^+^/Al^3+^ > 1). The high pressure model yielded an alkaline trend, similar to the anhydrous models, but follows along a trachybasalt-trachyte evolution path. The liquid compositions do not pass through the field of oceanic silicic rocks.

### Equilibrium partial melting models (SO_3_-free)

**Fig 4:**
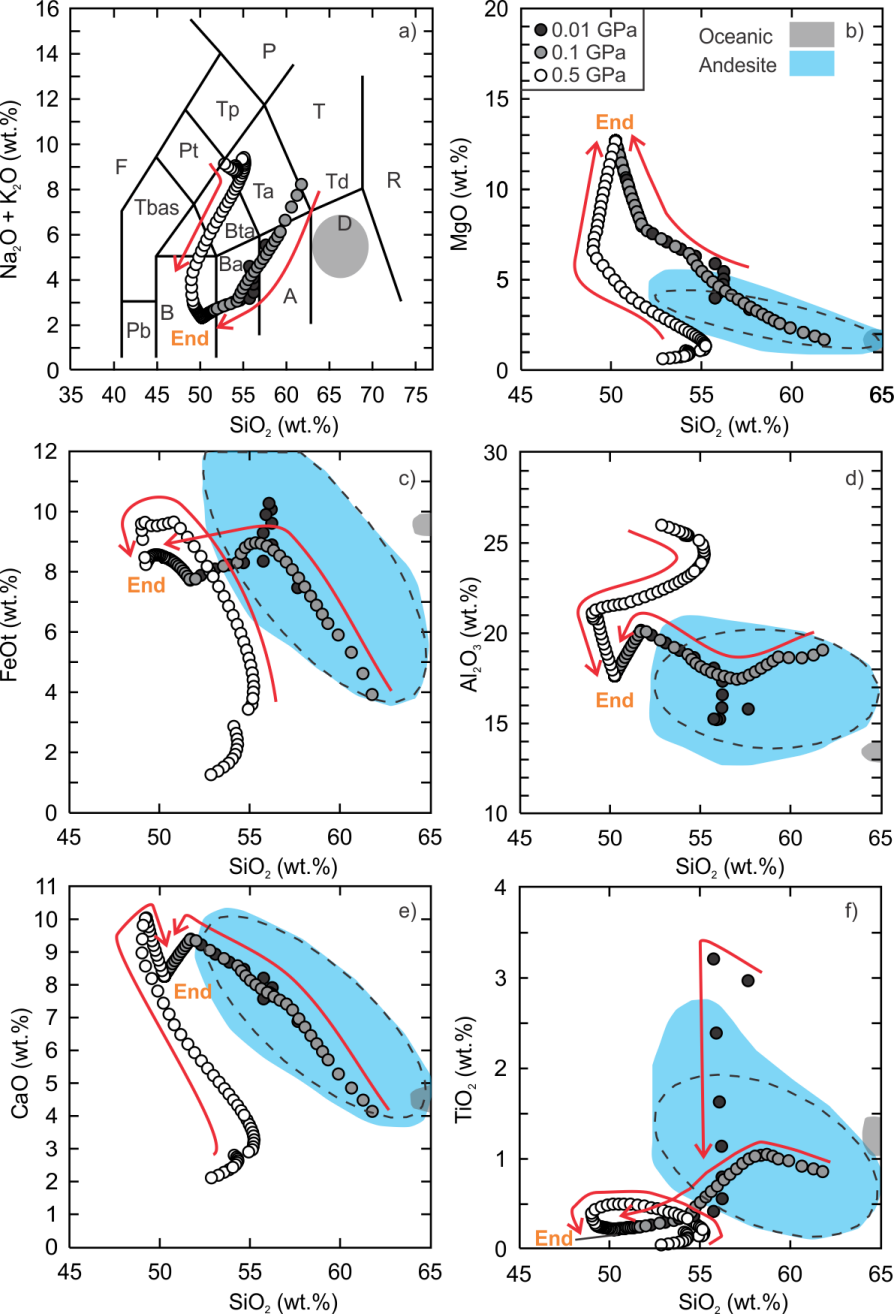
Results of Vega 2 hydrous (0.5 wt.%) equilibrium partial melting models. Andesite (blue field) data (S1 Table) are compiled from the GEOROC database (georoc.mpch-mainz.gwdg.de/georoc/Entry.html). The grey field is the range of silicic rocks from a mid-oceanic ridge setting [8]. All data are normalized to 100%. The calculated 95% confidence ellipses (dashed) are added to the field of terrestrial andesite. The details of panel are the same as Fig 2. Arrow is the direction of liquid evolution.

The equilibrium partial melting models produced liquid compositions that are andesitic (S4 and S5 Tables). The low and intermediate pressure hydrous partial melting models produced liquid compositions that are andesitic to trachydacitic (Fig 4). The intermediate pressure model produced the highest SiO_2_ content (~61 wt%) at a temperature of 970°C, melt fraction of ~0.2% and yielded a trachydacite liquid (Fig 4A). The low pressure model produced an andesitic liquid composition (SiO_2_ = ~57 wt%) representing a melt fraction of ~5%. The high pressure model (~10% melt fraction at 1010°C) did not produce a trachyandesite composition but has the lowest maximum SiO_2_ content (~55 wt%).

The anhydrous low pressure model requires a minimum temperature of 1130°C to generate a melt whereas the intermediate and high pressure models produced the first melts with temperatures of 1150°C and 1200°C respectively. The low pressure model produced the most silicic composition (SiO_2_ = 54 wt%) but is still broadly mafic. The intermediate pressure model only produced mafic liquids with the most evolved sample having SiO_2_ content of ~52.6 wt%. The high pressure model initially produced basanitic liquids (SiO_2_ < 47 wt%) before becoming basaltic.

### Fractional crystallization models (kieserite-adjusted)

**Fig 5:**
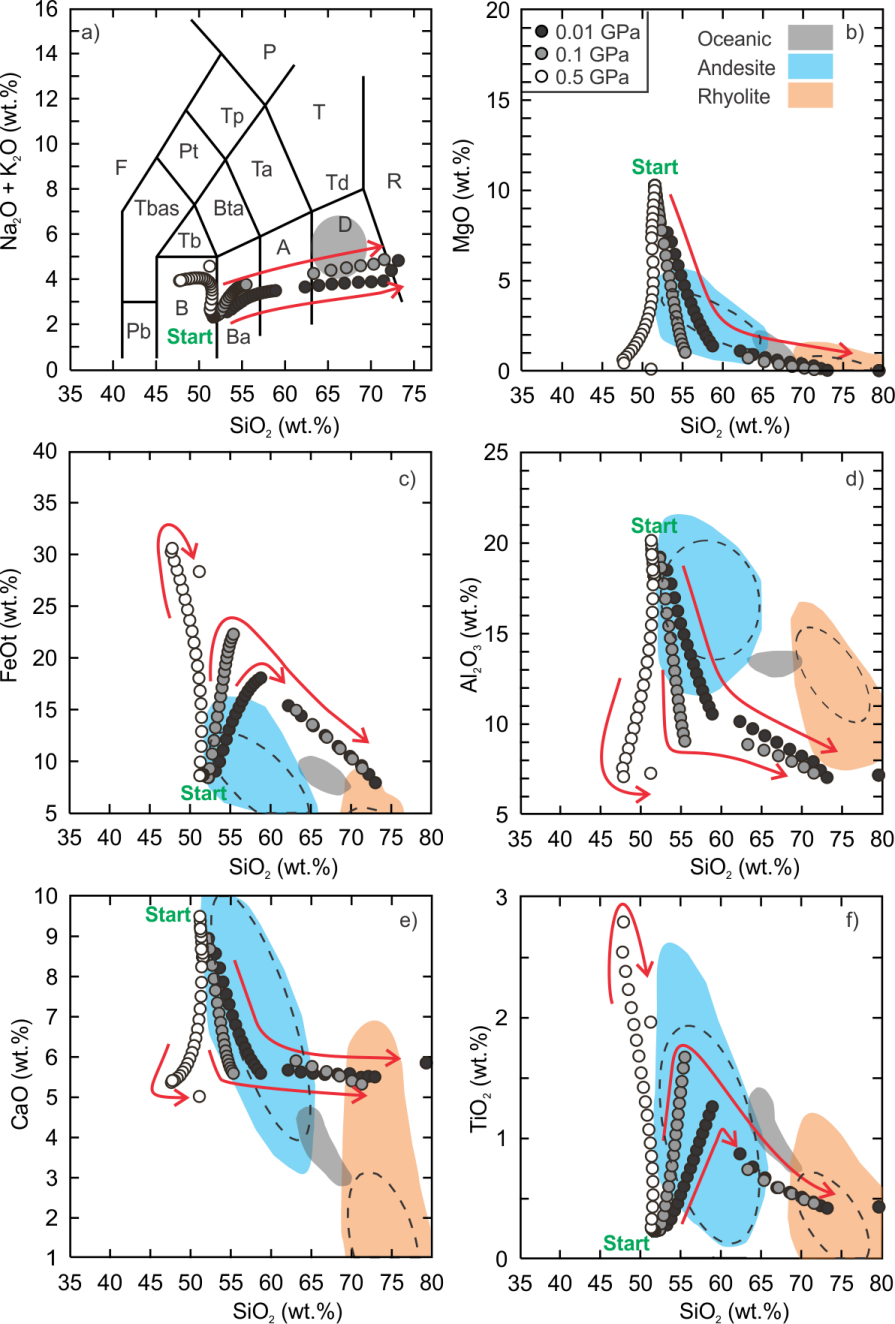
Results of Vega 2 anhydrous fractional crystallization models. The details of the figure are the same as Fig 2.

**Fig 6:**
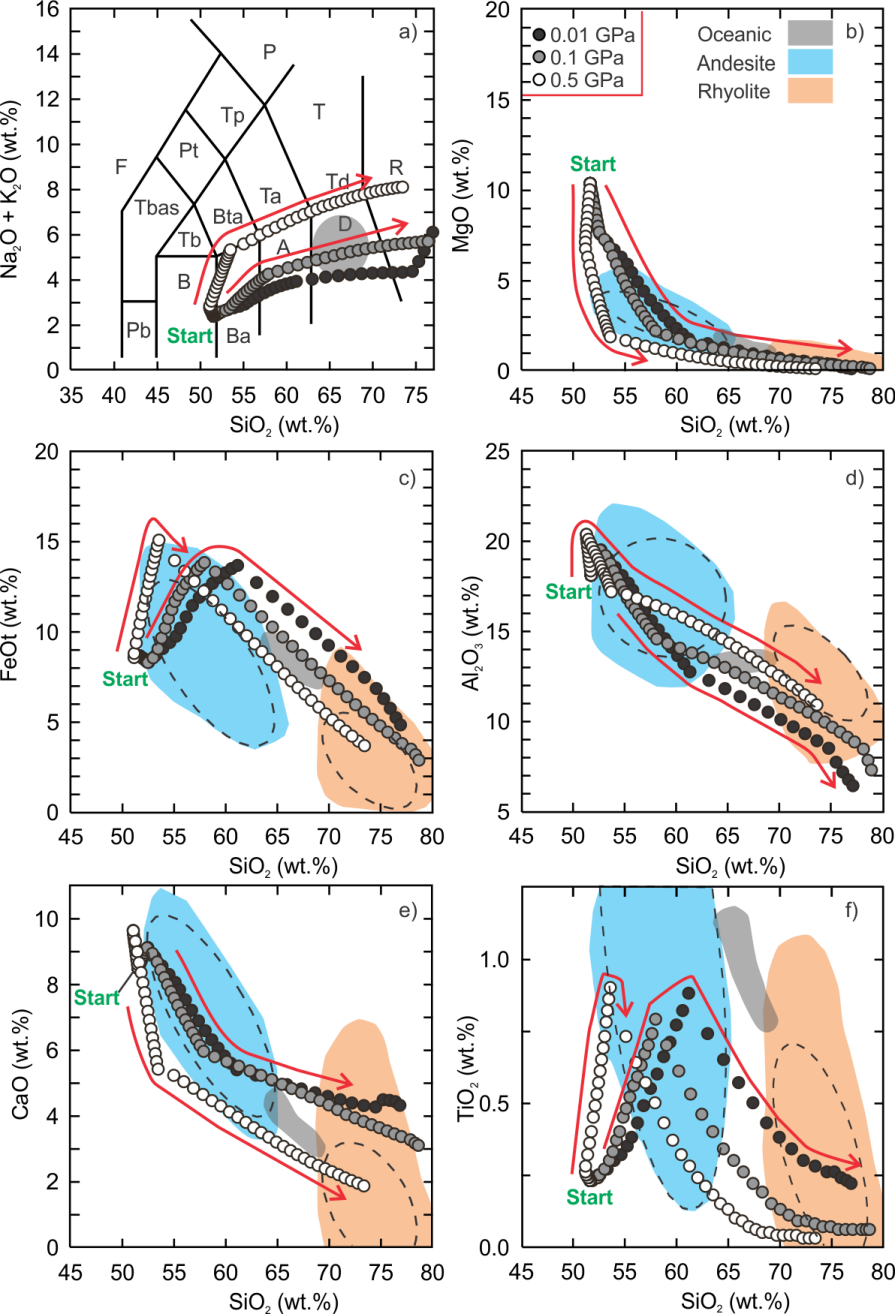
Results of Vega 2 hydrous (0.5 wt.% H_2_O) fractional crystallization models. The details of the figure are the same as Fig 2.

The fractionation models of the kieserite-adjusted composition also demonstrate that intermediate to silicic liquids can be generated. The anhydrous and hydrous fractionation modeling results can be found in the supplementary data tables (S6 and S7 Tables) and are presented in Figs 5 and 6.

The anhydrous, low (0.01 GPa) and intermediate (0.1 GPa) pressure models produced liquid compositions of basaltic andesite and dacite but there is a distinct silica gap between ~57 wt% and ~63 wt% for the 0.1 GPa models whereas the gap is smaller for the 0.01 GPa models (Fig 5). The gap is related to the onset of Ti-rich magnetite crystallization. The most silicic liquid compositions reach ~74 wt% SiO_2_ and just enter the field of rhyolite in the total alkalis vs. SiO_2_ classification diagram (Fig 5A). The dacitic low pressure-model residual liquid is 13.2% of the initial magma whereas the dacitic intermediate pressure-model liquid represents ~10.3%. Similar to the SO_3_-free models, the Al_2_O_3_ content of the low and intermediate pressure models is lower than that of terrestrial ferroan rhyolite. The lower bulk Al_2_O_3_ is related to the anhydrous nature of the model as more plagioclase crystallizes earlier (~9.5% of the crystallizing assemblage at 1220°C) than the hydrous models (~4.5% at 1190°C). The high pressure model did not yield a silicic composition (Fig 5).

In comparison to the anhydrous models, the entire series of hydrous models regardless of pressure yielded silicic liquid compositions (Fig 6). Moreover, the evolution curves do not have a silica gap and all curves pass through the fields of terrestrial ferroan andesite and ferroan rhyolite. The low to intermediate pressure liquid evolution curves, with the exception of TiO_2_, also pass through the field of oceanic silicic rocks. The silicic (SiO_2_ > 65 wt%) residual liquids for the low, intermediate and high pressure models represent 12.4% (1030°C), 20.2% (990°C) and 11.5% (950°C) of their initial magmas. The most significant difference between the anhydrous and hydrous models is the high pressure liquid evolution curve. Unlike the anhydrous high pressure model, the hydrous high pressure model yielded silicic compositions. The hydrous, low and intermediate pressure fractionation models produced broadly similar liquid compositions that are metaluminous and become peralkaline at higher SiO_2_ concentration (> 70 wt%). The high pressure model yielded higher total alkalis and lower CaO and TiO_2_ than the low to intermediate pressure models and follows the basaltic trachyandesite to trachydacite path before entering the rhyolite field.

### Equilibrium partial melting models (kieserite-adjusted)

**Fig 7:**
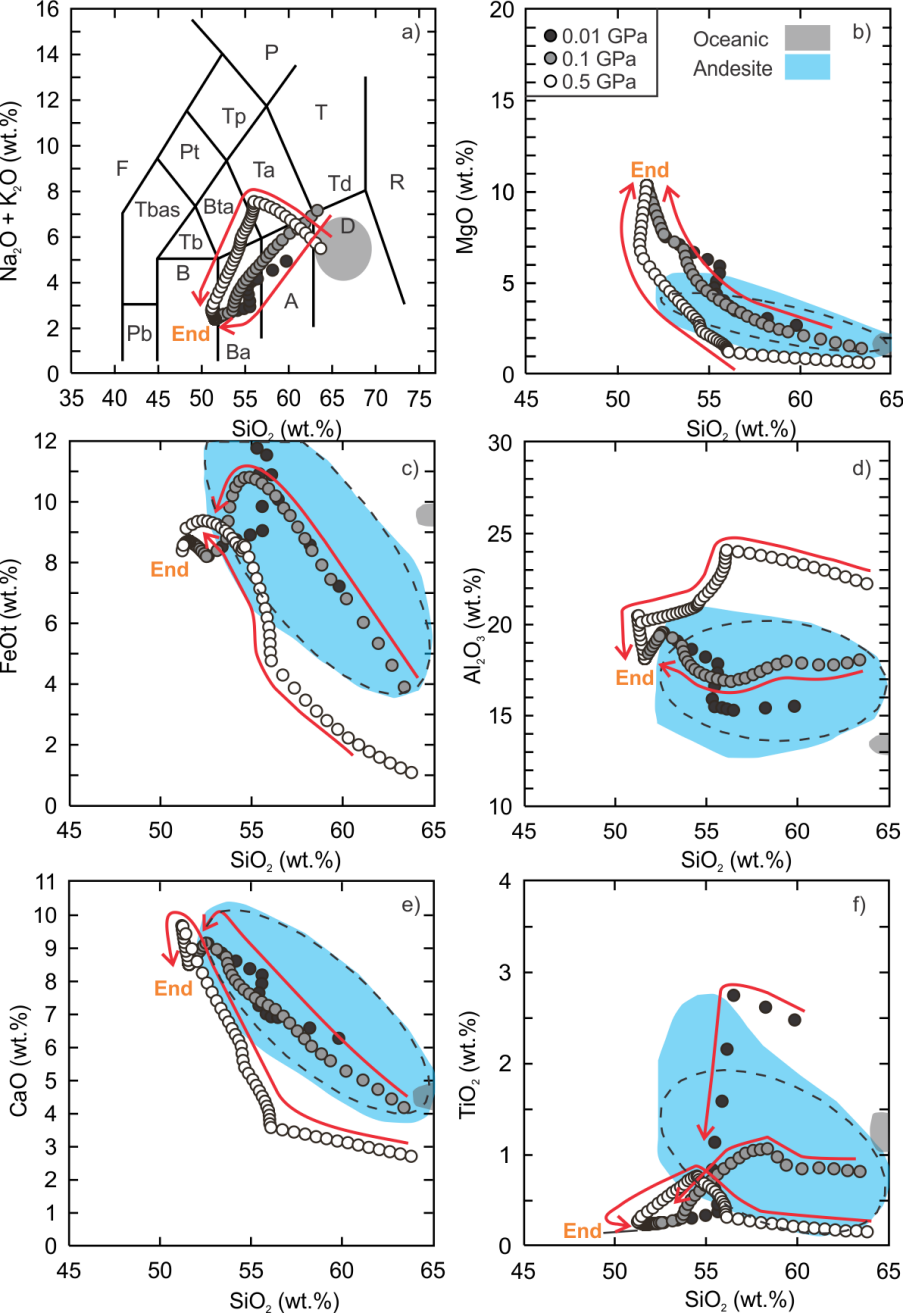
Results of Vega 2 hydrous (0.5 wt.%) equilibrium partial melting models. The details of the figure are the same as Fig 4.

The low to intermediate pressure hydrous equilibrium kieserite-adjusted partial melting models produced liquid compositions that are andesitic whereas the high pressure model reached dacitic compositions (Fig 7; S8 Table). The high pressure model yielded an andesite-dacite composition (~63.8 wt%) at a temperature of 810°C and melt fraction of ~4.5% (Fig 7A). However, the liquid compositions do not fall within the field of terrestrial ferroan andesite for CaO, FeOt, Al_2_O_3_ and TiO_2_ until a melt fraction of 12.8% (SiO_2_ = 55.1 wt%) is reached (1040°C). The low pressure model produced an andesitic liquid composition (SiO_2_ = ~57.6 wt%, 1070°C) representing a melt fraction of ~1.9%. The intermediate pressure model produced an andesitic liquid (SiO_2_ = ~60.3 wt%, 960°C) at a melt fraction of ~1%. The anhydrous models did not yield andesitic or silicic liquid compositions and therefore are not discussed.

### Effect of relative oxidation state

**Fig 8:**
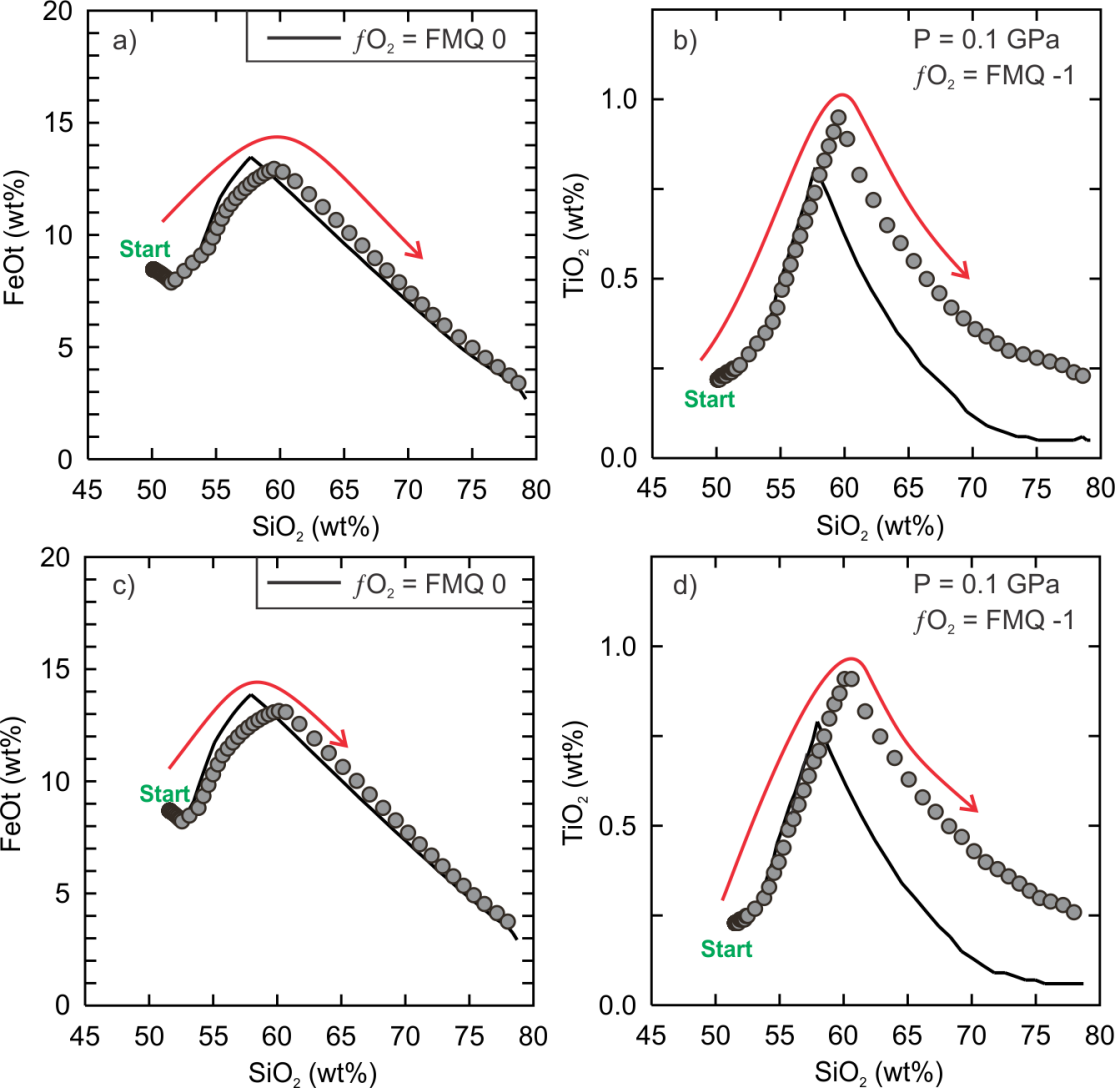
Comparison of the hydrous factional crystallization models at different relative oxidation states. (a) FeOt (wt%) and (b) TiO_2_ (wt%) vs. SiO_2_ (wt%) of the 0.1 GPa, hydrous SO_3_-free composition. The relative oxidation state is FMQ -1 for the data points (grey circles). The solid black curve is the original model at the FMQ buffer. (c) FeOt (wt%) and (d) TiO_2_ (wt%) vs. SiO_2_ (wt%) of the 0.1 GPa, hydrous kieserite-adjusted composition. The relative oxidation state is FMQ -1 for the data points (grey circles). The solid black curve is the original model at the FMQ buffer.

Basalts that erupt at within-plate tectonic settings tend to have magmatic relative oxidation states that range from the FMQ buffer to the WM buffer (FMQ 0 = WM +3.17) but it is closer to FMQ ± 1 for oceanic lithosphere [66,67]. In order to evaluate the effects of a more reducing relative oxidation state during fractional crystallization, additional models were run at FMQ -1 for the two hydrous Vega 2 compositions at 0.1 GPa (S9 Table). The results indicate that all elements, with the exception of TiO_2_ and FeOt, were unaffected by the change of relative oxidation state (Fig 8). The results show the liquid compositions will have marginally higher concentration of FeOt but significantly higher TiO_2_ than the models at the FMQ buffer. The results are expected as the crystallization of Fe-Ti oxide minerals (ilmenite, magnetite, ulvöspinel) is strongly influenced by the relative oxidation state of their parental magma [70].

Both models show that the spinel (Ti-rich magnetite) in the FMQ -1-model will crystallize (1010°C) 30°C lower than the FMQ 0-model (1040°C) but will have a higher ulvöspinel (Ti) component. The major difference is the initial amount of spinel that crystallizes. For the SO_3_-free composition, the FMQ -1-model indicates that spinel represents ~8.5% of the total amount of crystallizing phases (clinopyroxene, plagioclase and spinel) when the liquid temperature is 1010°C whereas in the case of the FMQ 0-model it represents ~16%. For the kieserite-adjusted composition, the amount of spinel crystallizing in the FMQ 0-model at 1040°C is ~17% but is only ~3.5% in the FMQ -1-model (at 1010°C). In other words, the more reducing relative oxidation state delays the onset of spinel crystallization and decreases the total amount that crystallizes.

### 3-Stage fractional crystallization models

The fractional crystallization models presented thus far are based on a 2-stage differentiation process. This section presents the results of a hydrous 3-stage differentiation process (S10 and S11 Tables). The 3-stage process assumes the Vega 2 rock composition is not primary and was derived by fractionation of olivine and probably clinopyroxene within the lower crust or uppermost mantle. The second and third stages of fractionation occur at 0.5 GPa and then 0.1 GPa (middle to upper crust). The liquid compositions used for the third stage models corresponds to the liquid compositions from the two (SO_3_-free and kieserite-adjusted) 0.5 GPa models (FMQ 0) at 1220°C (S9 and S10 Tables). The amount of crystals removed from the liquid at 1220°C for the SO_3_-free model is ~35% (23.4% orthopyroxene, 2.4% clinopyroxene, and 9.5% plagioclase) whereas amount of crystals removed in the kieserite-adjusted models is ~30% (15.9% orthopyroxene, 4.4% clinopyroxene, and 9.6% plagioclase). Each model was conducted using relative oxidation states at the FMQ buffer and FMQ -1. The relative oxidation state of the 0.5 GPa models does not influence the resultant liquid compositions because the Fe-Ti oxide minerals do not crystallize before 1220°C.

**Fig 9:**
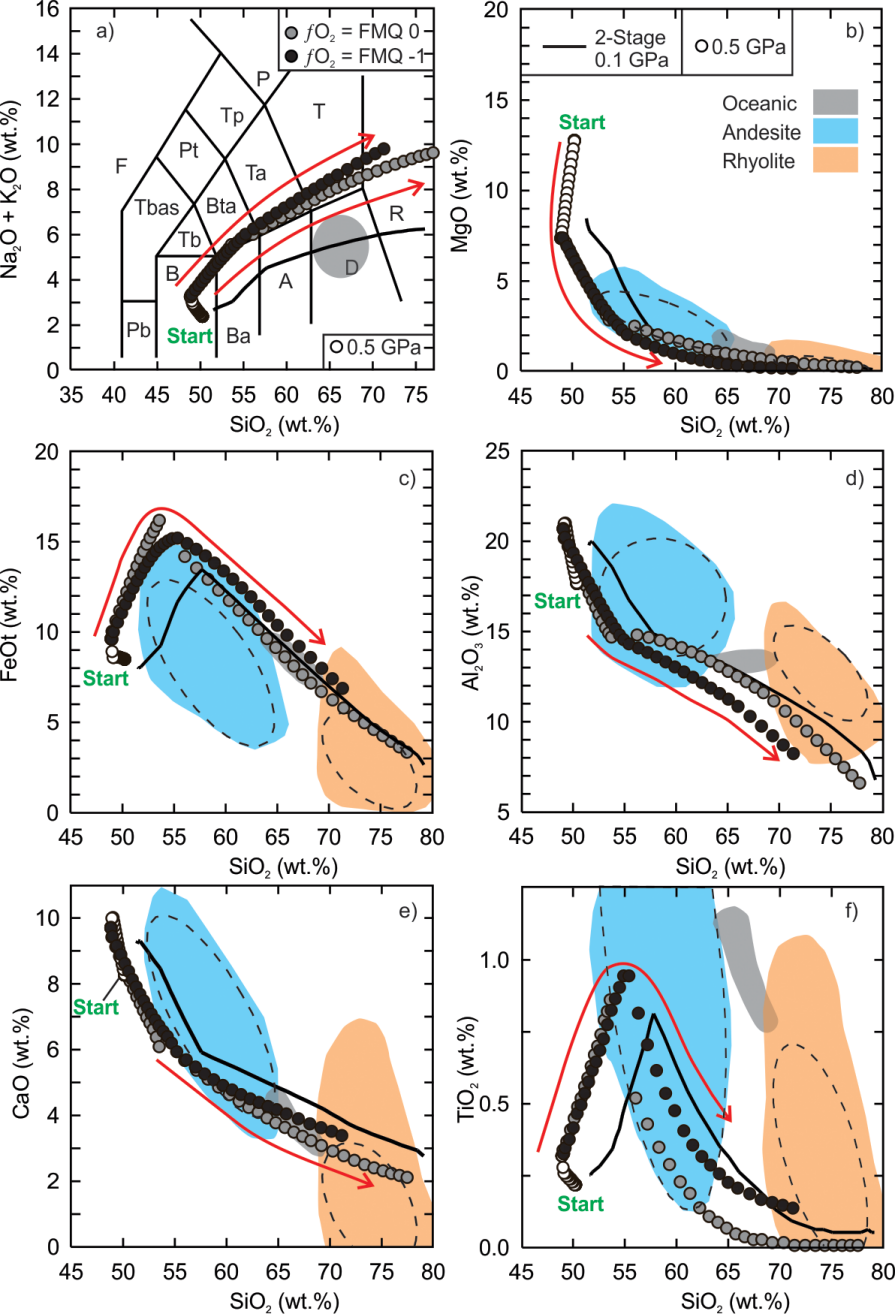
Results of 3-stage, SO_3_-absent Vega 2 hydrous (0.5 wt% H_2_O) fractional crystallization models. The details of the figure are the same as Fig 2.

The results of the hydrous SO_3_-free models are shown in Fig 9 along with the hydrous 2-stage fractionation model (solid line). The 3-stage models, regardless of relative oxidation state, can yield highly silicic liquids that are within the range of ferroan rhyolites. The most significant difference between the 2-stage and 3-stage models at the FMQ buffer is the increase in the total Na_2_O and K_2_O contents. The 3-stage model shows the alkalis will reach ~8 wt% at 70% wt% SiO_2_ whereas they are ~6 wt% in the 2-stage model. Furthermore, CaO is ~1 wt% lower at 70% SiO_2_ in the 3-stage model. The only element that may be outside the range of ferroan rhyolite is TiO_2_. The TiO_2_ contents reach exceptionally low values (<0.05) before ~70 wt% SiO_2_. In addition to TiO_2_, the results from the 3-stage FMQ -1-model differ from the 3-stage FMQ 0-model with respect to the Al_2_O_3_ and SiO_2_ contents (lower).

**Fig 10:**
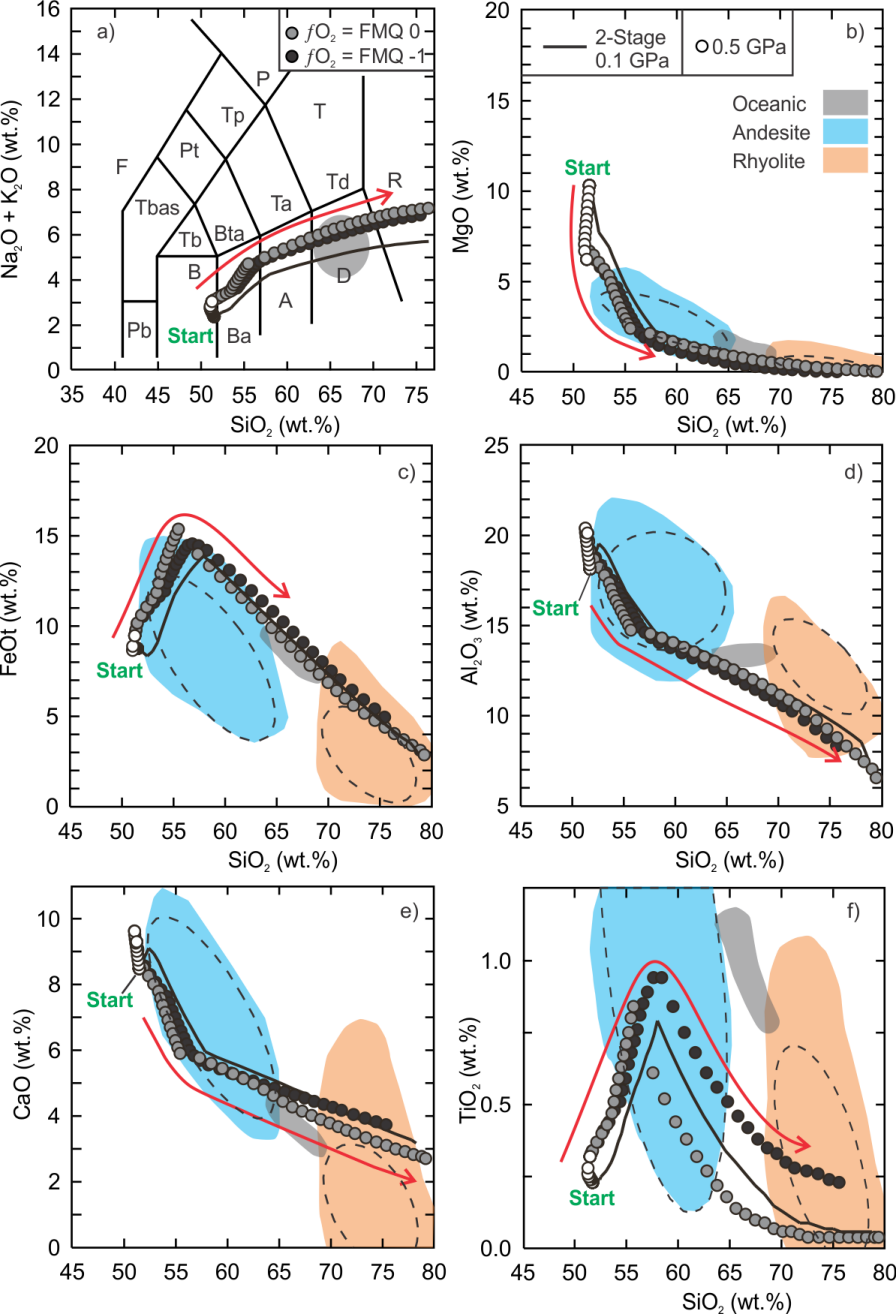
Results of 3-stage, kieserite-adjusted Vega 2 hydrous (0.5 wt.% H_2_O) fractional crystallization models. The details of the figure are the same as Fig 2.

The results of the hydrous kieserite-adjusted models are shown in Fig 10 along with the hydrous 2-stage fractionation model (solid line). Both of the 3-stage models indicate that highly silicic liquids similar to ferroan rhyolite can be generated. Moreover, the liquid evolution curve passes through the range of oceanic-silicic rocks for all elements for the exception of TiO_2_. Similar to the 3-stage SO_3_-free models, the total alkalis are higher than the 2-stage model but only by ~1 wt%. All other elements for the exception of TiO_2_ (lower) are similar to the 2-stage model. The results from the 3-stage FMQ -1-model only differ with respect to the TiO_2_ content (higher) of the 3-stage FMQ 0-model.

## Discussion

### Silicic liquids derived by fractional crystallization

The Rhyolite-MELTS modeling results indicate that intermediate to silicic liquids can be derived from a parental magma similar to the SO_3_-free and kieserite-adjusted compositions of the Vega 2 rock. The hydrous and anhydrous, relatively reducing or oxidizing, 2-stage or 3-stage, high or low pressure, fractionation models were able to produce a range of silicic compositions from andesite to rhyolite. However, the fractionation results do not necessarily imply that all of the models are appropriate for the generation of silicic liquids on Venus. This section addresses the prospects of the different fractionation models.

The wet-dry dichotomy of Venus is a problem for evaluating the water content of magma derived from the mantle. Although the surface temperature is high and water is not currently present, there is evidence (atmospheric water, high D/H ratio) to suggest there was water in the geological past. Consequently, it is very likely that the mantle of Venus contained and still contains volatile elements (e.g. H_2_O, CO_2_, SO_2_, H_2_S, Cl-rich molecules, F-rich molecules) that are released in gaseous form during volcanism. Thus the hydrous fractionation models are probably more ‘realistic’ than the anhydrous models [49].

The relative oxidation state of a magma derived from the Venusian mantle is unknown. At the moment there is no way to verify which relative oxidation stage used in the models was closer to the actual situation that lead to the Vega 2 rock. However, based on the bulk TiO_2_ it is possible that the Vega 2 rock had a lower oxidation state than the Venera 13 and Venera 14 rocks [49]. It is suggested that terrestrial basalt with a TiO_2_/Fe_2_O_3_ ratio of 0.5 is indicative of a reducing mantle source whereas rocks with a ratio of 1.0 were derived from an oxidized mantle source [71]. Assuming a Fe^3+^/Fe^2+^ ratio of 0.15 for the Venusian basalt then it appears that the Venera 13 and Venera 14 rocks have a TiO_2_/Fe_2_O_3_ ratio closer to 1.0 whereas the Vega 2 rock is closer to 0.5 [72]. This does not confirm the Vega 2 magma had a relatively reducing oxidation state only that it is possible. Perhaps the most important implication of the FMQ -1-models is that they yield silicic liquids with higher TiO_2_ contents.

Multiple magmatic stages are interpreted for the genesis of silicic plutonic and volcanic rocks on Earth [57,73,74]. The 2-stage and 3-stage models presented in this paper are equally plausible geological scenarios that yield silicic compositions and it is likely that they both operate in the crust of Venus. The principle difference between the 2- and 3-stage models is that the total alkalis are generally higher in the 3-stage models although the 2-stage high pressure kieserite-adjusted model produces high total alkalis as well. The higher alkali content is a consequence of the additional step that removes more (30–35%) non-alkali minerals (pyroxenes and Ca-plagioclase) from the liquid prior to the final stages of fractionation. The higher total alkalis content is more consistent with alkalic ferroan silicic rocks (Na_2_O+K_2_O > 7 wt% at SiO_2_ = 70 wt%) at within-plate settings (continental large igneous provinces) but there are many silicic rocks from a similar tectonic setting that have lower total alkali compositions [47,75,76].

From a compositional point of view, the lack of P_2_O_5_ in the starting material does not pose a significant problem with the fractionation results as it pertains to the CaO content of the residual liquids. The presence of P_2_O_5_ would permit apatite [Ca_5_(PO_4_)_3_(F,Cl,OH)] to crystallize in the model and likely reduce the amount of CaO in the liquid composition by ~0.2 wt% assuming a whole rock P_2_O_5_ content of 0.25 wt% at a liquid equal to ~65 wt% SiO_2_. Moreover, it is possible that other Ca-rich non-silicate minerals like fluorite play a role in calcium fractionation but are not factored into the models [77].

Overall the hydrous fractionation models are more plausible than the anhydrous models. The various pressure (0.5 GPa, 0.1 GPa, 0.01 GPa), oxidation state (FMQ 0 or FMQ -1) and stages of differentiation are important for generating specific liquid compositions (e.g. high alkali and high TiO_2_ contents) but they are not fixed parameters. In reality the magmatic conditions will vary and, as demonstrated, lead to silicic liquids with slightly different compositions. In the strictest of terms the silicic liquid compositions derived from the 2- and 3-stage kieserite-adjusted models are probably the most similar to terrestrial silicic rocks.

### Silicic liquids derived by equilibrium partial melting

The results of the equilibrium partial melting models can yield silicic liquids (SiO_2_ ≈ 60 wt%) but did not reproduce highly silicic compositions (SiO_2_ > 70 wt%). The intermediate pressure (0.1 GPa) hydrous models (SO_3_-free and kieserite-adjusted) produced andesite-dacite compositions (SiO_2_ ≈ 57 wt% to 63 wt%). The hydrous low and high pressure models produced andesitic compositions that are similar to terrestrial rocks but the minimum temperature to produce a melt is 950°C (0.1 GPa, kieserite-adjusted). In comparison, the anhydrous models did not produced silicic liquids and require a very high temperature to generate the first melt (≥ 1130°C). It is highly unlikely that the thermal regime required to generate the initial anhydrous melts could be sustained in the upper to middle crust of Venus as the transfer of heat from the injection of mafic magma to melt andesitic crust is inefficient [2,62,78]. The mantle potential temperature (*T*_P_) estimate of the primary magma of the Vega 2 composition is estimated to be either ~1400°C or ~1780°C [48,51]. The lower *T*_P_ is similar to the ambient mantle conditions of the modern Earth whereas the higher estimate is similar to the thermal regime of terrestrial Archean komatiites [53]. Basaltic magma derived from a primary melt in the lower *T*_P_ regime is unlikely to create high enough temperatures to melt basaltic crust [79,80]. Primary ultramafic magmas produced from a high *T*_P_ regime may melt mafic lower crust if it were hydrous and magmatism was sustained for thousands of years [81]. Ultramafic lavas probably erupted during the development of the early Venusian crust in a similar manner as Earth when mantle temperatures were likely higher [82–87]. However, the calculated primary melt composition from the Venera 14 landing site, a possible proxy for the Vega 2 primary melt, is picritic and likely had an eruption temperature of ~1300°C [48,52]. Although it is possible that crustal melting may occur due to the injection of an ultramafic magma into hydrous basaltic crust, less likely if the magma is basaltic, it is probably a process that only produces very minor volumes of intermediate compositions [40]. It is more likely that intermediate to silicic liquids are produced by partial melting of older (earlier formed) intermediate or silicic volatile-bearing crustal rocks that originally formed by fractional crystallization.

### Implications for the upper crust of NE Aphrodite Terra

The modeling results presented in this study only indicate that silicic liquids, under the scenarios outlined, can be derived from a parental magma composition similar to that analyzed at the Vega 2 landing site. However, the possibility that silicic magmas and lavas were emplaced has important implications for the structure of the upper crust of NE Aphrodite Terra and Venus in general. For example, the modeling results indicate that it is possible the inferred Venera 8 composition may be silicic and the identified pyroclastic deposits may have formed by intermediate to silicic volatile-rich magmas [19,24]. This section discusses the tectonic, rheological and geological significance of silicic rocks within Venusian crust.

An extensional tectonic setting is the most likely environment for the formation of silicic liquids by fractional crystallization in the shallow (≤ 5 km depth) crust. Tensional plate stress could be due to mantle upwelling or passive rifting. Petrogenetically related bimodal plutonic-hypabyssal-volcanic systems within the shallow continental crust are observed on Earth at continental rifts, Iceland and large igneous provinces [88–91]. In most cases the rock assemblages are interpreted to represent dynamic magma chambers where mafic or ultramafic cumulus zones are located below more evolved (i.e. silicic) units. Some of the layered complexes have direct evidence that the upper silicic magmas were the source of feeder dykes that erupted on the surface [92]. It is very likely that silicic igneous rocks, either volcanic or plutonic, derived from basaltic parental magmas are present within the crust of Venus. If the development of silicic rocks on Venus is analogous to terrestrial large igneous provinces then the volume within Venusian crust should be ≤ 10% and less dense overall than if it were pure basaltic crust [93,94].

The origin of pancake (broad, flat and circular) domes on Venus is debated but they are interpreted to be the remnants of viscous silicic lava [17,25]. Whether a silicic magma erupts or not is dependent on a number of parameters including: viscosity, volatile content, depth and repeated magma injection [95–97]. The viscosity of lava is dependent on a number of factors which include crystal content, temperature, composition, and the rheology of the country rock [98,99]. The volatile content of lava/magma will also help to reduce viscosity and thus affect the likelihood of eruption although the rheological properties of the country rock, crystal content, and chemical composition play a significant role [98,99]. The crystal-free viscosity estimates for the silicic liquids in the 0.1 GPa anhydrous models (10^6^ poise) are higher at equivalent temperature (1000°C) than for the hydrous models (10^3.5^–10^4.2^ poise). Although a crystal-free liquid is not likely to exist, the difference in viscosity estimates between the anhydrous and hydrous models suggests that the compositions generated by the hydrous model liquids will initially have a higher likelihood of erupting before other parameters (e.g. crystallinity, composition, tectonic setting) influence the system. Moreover, the temperature at which the silicic liquids form is high (≥ 800°C) but within the range expected for within-plate settings [100,101]. Therefore, although it is possible the pancake domes are formed by viscous silicic lavas, silicic volcanic rocks on Venus would not necessarily be viscous nor be restricted in their eruption style and structure.

The primary focus of this paper is on the initial generation of silicic liquids on Venus. However, once silicic rocks have formed they can be reworked during subsequent tectonomagmatic episodes (e.g. partial melting and compressional tectonics). The geological complexity of highland terranes suggests they represent regions of tectonically remobilized crust [102–110]. The consequence of crustal remobilization would be the formation of the second generation silicic rocks and the development of metamorphic rocks. The second generation silicic rocks may act as preferred zones of deformation as plagioclase-poor rocks are weaker than plagioclase-bearing rocks [111]. In other words, from a terrestrial point of view, it is possible that highland terranes could be a Venus analogue of granite-greenstone belts that formed during the early tectonic evolution of Earth [110,112].

## Conclusions

Petrological modeling of the basalt analyzed at the Vega 2 landing site indicates that intermediate to silicic liquids can be generated on Venus under reasonable geological conditions by fractional crystallization and equilibrium partial melting. The hydrous fractional crystallization 2- and 3-stage models yield liquid compositions that best resemble terrestrial silicic rocks that are found at continental rifting sites or within large igneous provinces. The hydrous partial melting models at low to intermediate pressure can produce andesitic liquids but requires relatively high temperatures (≥ 950°C) to generate the first liquids. The anhydrous partial melting models can produce basaltic andesite compositions but at a very high temperature (≥ 1130°C) that is unlikely to be frequent or sustained. Although silicic rocks are not definitively identified on the surface of Venus, it is probable that they exist and represent a small but important component of the Venusian crust.

## Supporting information

S1 Table Major elemental compositions of ferroan andesite (metaluminous) and rhyolite (metaluminous and peralkaline). The results were compiled from the GEOROC database (http://georoc.mpch-mainz.gwdg.de/georoc/). Only results with < 2.5 wt% loss on ignition were used. The andesites samples are defined by SiO_2_ >52 wt% by <64 wt% whereas the rhyolites have SiO_2_ >69 wt%. All results were recalculated to 100%.
(XLS)

S2 Table Results of 2-stage anhydrous fractional crystallization modeling of the Vega 2 basalt (SO_3_-free). The results presented in this file are the raw output data generated by Rhyolite-MELTS for dry fractional crystallization of Vega 2 basalt (SO_3_-free) listed in Table 1. The relative oxidation state is fixed to the FMQ buffer, pressures of 0.01, 0.1 and 0.5 GPa. The liquid compositions are the basis of the model curves presented in Fig 2 of the text.
(XLS)

S3 Table Results of 2-stage hydrous fractional crystallization modeling of the Vega 2 basalt (SO_3_-free). The results presented in this file are the raw output data generated by Rhyolite-MELTS for wet fractional crystallization of Vega 2 basalt (SO_3_-free) listed in Table 1. The relative oxidation state is fixed to the FMQ buffer, pressures of 0.01, 0.1 and 0.5 GPa and initial water content of 0.5 wt% were used. The liquid compositions are the basis of the model curves presented in Fig 3 of the text.
(XLS)

S4 Table Results of hydrous partial melting modeling of the Vega 2 basalt (SO_3_-free). The results presented in this file are the raw output data generated by Rhyolite-MELTS for wet partial melting of Vega 2 basalt (SO_3_-free) listed in Table 1. The relative oxidation state is fixed to the FMQ buffer, pressures of 0.01, 0.1 and 0.5 GPa and initial water content of 0.5 wt% were used. The liquid compositions are the basis of the model curves presented in Fig 4 of the text.
(XLS)

S5 Table Results of anhydrous partial melting modeling of the Vega 2 basalt (SO_3_-free). The results presented in this file are the raw output data generated by Rhyolite-MELTS for dry partial melting of Vega 2 basalt (SO_3_-free) listed in Table 1. The relative oxidation state is fixed to the FMQ buffer, pressures of 0.01, 0.1 and 0.5 GPa and initial water content of 0 wt% were used.
(XLS)

S6 Table Results of 2-stage anhydrous fractional crystallization modeling of the Vega 2 basalt (kieserite-adjusted). The results presented in this file are the raw output data generated by Rhyolite-MELTS for dry fractional crystallization of Vega 2 basalt (kieserite-adjusted) listed in Table 1. The relative oxidation state is fixed to the FMQ buffer, pressures of 0.01, 0.1 and 0.5 GPa. The liquid compositions are the basis of the model curves presented in Fig 5 of the text.
(XLS)

S7 Table Results of 2-stage hydrous fractional crystallization modeling of the Vega 2 basalt (kieserite-adjusted). The results presented in this file are the raw output data generated by Rhyolite-MELTS for wet fractional crystallization of Vega 2 basalt (kieserite-adjusted) listed in Table 1. The relative oxidation state is fixed to the FMQ buffer, pressures of 0.01, 0.1 and 0.5 GPa and initial water content of 0.5 wt% were used. The liquid compositions are the basis of the model curves presented in Fig 6 of the text.
(XLS)

S8 Table Results of hydrous partial melting modeling of the Vega 2 basalt (kieserite-adjusted). The results presented in this file are the raw output data generated by Rhyolite-MELTS for wet partial melting of Vega 2 basalt listed in Table 1. The relative oxidation state is fixed to the FMQ buffer, pressures of 0.01, 0.1 and 0.5 GPa and initial water content of 0.5 wt% were used. The liquid compositions are the basis of the model curves presented in Fig 7 of the text.
(XLS)

S9 Table Results of 2-stage hydrous fractional crystallization modeling of the Vega 2 basalt (SO_3_-free and kieserite-adjusted) at FMQ -1. The results presented in this file are the raw output data generated by Rhyolite-MELTS for wet fractional crystallization of Vega 2 basalt listed in Table 1. The relative oxidation state is fixed to the FMQ -1 and pressure of 0.1 GPa and initial water content of 0.5 wt% were used. The liquid compositions are the basis of the model curves presented in Fig 8 of the text.
(XLS)

S10 Table Results of 3-stage hydrous fractional crystallization modeling of the Vega 2 basalt (SO_3_-free). The results presented in this file are the raw output data generated by Rhyolite-MELTS for wet fractional crystallization of Vega 2 basalt (SO_3_-free) listed in Table 1. The relative oxidation state is set to the FMQ buffer and FMQ -1 and pressure = 0.1 GPa. The starting composition for this model was taken from S2 Table at 0.5 GPa and 1220°C. The liquid compositions are the basis of the model curves presented in Fig 9 of the text.
(XLS)

S11 Table Results of 3-stage hydrous fractional crystallization modeling of the Vega 2 basalt (kieserite-adjusted). The results presented in this file are the raw output data generated by Rhyolite-MELTS for wet fractional crystallization of Vega 2 basalt (kieserite-adjusted) listed in Table 1. The relative oxidation state is set to the FMQ buffer and FMQ -1, and pressure = 0.1 GPa. The starting composition for this model was taken from S6 Table at 0.5 GPa and 1220°C. The liquid compositions are the basis of the model curves presented in Fig 10 of the text.
(XLS)
